# Prediction of Flight Status of Logistics UAVs Based on an Information Entropy Radial Basis Function Neural Network

**DOI:** 10.3390/s21113651

**Published:** 2021-05-24

**Authors:** Qin Yang, Zhaofa Ye, Xuzheng Li, Daozhu Wei, Shunhua Chen, Zhirui Li

**Affiliations:** School of Mechanical Engineering, Hefei University of Technology, Hefei 230009, China; 2019110116@mail.hfut.edu.cn (Z.Y.); 2020110193@mail.hfut.edu.cn (X.L.); weidaozhu@126.com (D.W.); shchen@hfut.edu.cn (S.C.); lizhirui202105@163.com (Z.L.)

**Keywords:** internet of things, logistics drones, RBF neural network, information entropy, nonlinear system

## Abstract

Aiming at addressing the problems of short battery life, low payload and unmeasured load ratio of logistics Unmanned Aerial Vehicles (UAVs), the Radial Basis Function (RBF) neural network was trained with the flight data of logistics UAV from the Internet of Things to predict the flight status of logistics UAVs. Under the condition that there are few available input samples and the convergence of RBF neural network is not accurate, a dynamic adjustment method of RBF neural network structure based on information entropy is proposed. This method calculates the information entropy of hidden layer neurons and output layer neurons, and quantifies the output information of hidden layer neurons and the interaction information between hidden layer neurons and output layer neurons. The structural design and optimization of RBF neural network were solved by increasing the hidden layer neurons or disconnecting unnecessary connections, according to the connection strength between neurons. The steepest descent learning algorithm was used to correct the parameters of the network structure to ensure the convergence accuracy of the RBF neural network. By predicting the regression values of the flight status of logistics UAVs, it is demonstrated that the information entropy-based RBF neural network proposed in this paper has good approximation ability for the prediction of nonlinear systems.

## 1. Introduction

The emergence of logistics UAVs solves the problem of goods transportation in remote and underdeveloped areas, which plays an important role in promoting the construction and development of the global economy [1,2]. In addition to this, high level urban traffic conditions are an issue for public management and in order to achieve precise and accurate traffic studies, the real traffic flow conditions in urban areas can be evaluated based on videos acquired by UAVs [3]. However, many issues have not been solved for the use of logistics UAVs, for example, short battery life, limited load, low payload, high operating cost, and the fact they are easily affected by the weather [4,5]. Part of the pulling force generated by an UAV is used to lift and stabilize the weight of fuselage and cargo, to resist wind and to complete forward and backward, left and right roll operations. Under different loads, the measurement of flight stability of logistics UAV needs to be solved. Therefore, the study of UAV flight state prediction is crucial.

Scholarly research on UAV flight state prediction is currently a very hot topic, which can be divided into two categories according to the different principles of UAV prediction: modeled prediction techniques and modeless prediction techniques.

Modeled prediction techniques predict the target trajectory based on the established target equations of motion combined with the predicted current state of the target. Porretta et al. [6] integrated the wind speed and lateral braking force of the aircraft to build a performance model of the aircraft, and used the aircraft navigation and intention information as the input parameters of the model to predict the flight trajectory based on the aircraft flight plan calculation. Prandini et al. [7] used Brownian motion to describe the uncertainty and modeled the aircraft motion with uncertainty, assuming that the aircraft maintains a uniform flight speed, through which the aircraft trajectory is predicted, and then the airspace complexity is evaluated based on the trajectory prediction results. Since the flight plan of the aircraft is not considered in the modeling process, the timeliness of the method is limited. Benavides et al. [8] proposed an UAV trajectory prediction algorithm based on a kinematic model from two perspectives of aircraft horizontal profile and altitude profile, and integrated factors such as fuel consumption and planned flight time, after which, the flight process was simulated by simulation experiments, and it was concluded that the fuel consumption during flight could be reduced and the operation quality of UAV could be improved by trajectory prediction.

It can be seen that the target motion models used by the modal trajectory prediction technique are usually based on certain assumptions, i.e., under certain ideal conditions under which they are built. Since the factors affecting the target trajectory are very complex in the actual operating environment, it is extremely difficult to accurately model this considering all the influencing factors, and the more accurate the modeling is, the less generalizable the model built often is, so multiple motion models may need to be built to match the motion states of the target at each time. Unlike the model trajectory prediction technique, the modeless trajectory prediction technique treats the trajectory data as time series and abstracts the trajectory prediction problem as a time-series prediction problem, thus avoiding the problem of modeling complex motions. Some of the model-free trajectory prediction techniques only use several trajectory points before the current moment to predict future trajectories, such as the asymptotically optimal target trajectory prediction algorithm proposed by Chen et al. [9]. These trajectory prediction methods use less data, less computation, and high generalizability, but they do not make full use of the historical trajectory information of the target, and only predict the trajectory based on the information of several trajectory points before the current moment, and when the predicted trajectory of the target is complex, these methods may not accurately grasp its future motion trend. The historical trajectory of the motion target contains its unique motion law, and some model-free trajectory prediction techniques can improve the accuracy of trajectory prediction by using a large number of historical trajectories of the predicted target, and the more representative trajectory prediction methods include trajectory prediction methods based on Gaussian mixture model, trajectory prediction methods based on neural network and trajectory prediction methods based on Markov model, etc. Wiest et al. [10] used a Gaussian mixture model for vehicle trajectory prediction, converting the trajectory data from a position coordinate representation to a representation by velocity and yaw rate, and bringing the above features into the Gaussian mixture model for training, which allows the prediction model to update more quickly when the direction of travel of the target vehicle is predicted to change. Qiao et al. [11] proposed an adaptive parametric trajectory prediction model based on Hidden Markov Model, which considers the velocity variation of the predicted target and improves the prediction efficiency of the algorithm by density-based processing of the trajectory division. Sanchez-Garcia et al. [12] accelerated the convergence speed of the particle swarm algorithm by improving the specific range of the environmental search to obtain the optimal trajectory faster. Yuan et al. [13] improved the robustness and convergence speed of the algorithm by constructing a geometric space structure to guide the particle swarm motion. Hafez et al. [14] proposed a method that combines control parameterization and time discretization with particle swarm optimization to solve the trajectory optimization problem. Song et al. [15] proposed a new multimodal delayed particle swarm optimization algorithm that reduces the occurrence of local convergence of the trajectory planning and improves the trajectory planning performance.

In general, the advantage of the modeless trajectory prediction technology is that the prediction target itself does not need to be modeled precisely, and it mainly starts from the temporal sequence of trajectory information, and learns the motion law of the prediction target by means of data mining and fitting, so as to achieve the prediction effect.

The development of RBF neural network provides a new method for industrial process modeling and control. Because of its simple structure and strong approximation ability, RBF neural network has been widely used in practical application and theoretical research [16,17,18,19]. RBF design is mainly about structure design and learning algorithm design. The design of algorithm has become more and more mature, but the structure design is still an unsolved problem, i.e., the radial basis function. Radial basis function neural network is a kind of efficient feedforward neural network, which has the best approximation performance and global optimal performance superior to other feedforward neural networks, and has simple structure and fast training speed. The RBF neural network is trained by using of experimental input/output data [18]. Due to the small number of input samples, the accuracy of the final convergence decreased. Aiming at the structural change and optimization of RBF neural network in training, Platt [19] proposed a kind of resource allocation network (RAN), which can increase hidden layer neurons, according to the complexity of processing objects. However, the continuous increase will lead to the decrease of calculation efficiency. Lu et al. [20] proposed a kind of minimum resource allocation (MRA network, MRAN), which can actively increase, or reduce the number of neurons in the hidden layer during network training. This has been approved by the structural design method of the neural network [21]. The adjustment of parameters has not been considered after the structural change, therefore, the convergence speed is slow. Lian et al. [22] proposed a self-organizing RBF (SORBF) neural network RBF. RBF is the basis of structural adjustment by continuously approaching the expected error, but it does not take into account the connection information between the hidden layer and the output layer, and the reset of parameters after structural adjustment. The training accuracy is therefore not high, and the convergence time is long. Huang et al. [23] proposed a kind of extended growing and pruning RBF (GGAP-RBF) neural network, by calculating the importance of hidden layer nodes. The nodes are added or deleted, but the initial value setting of network structure parameters needs to refer to the overall sample data, which is not suitable for online learning. Based on the above, it can be seen that structural optimization of RBF neural networks is still an open problem, especially the convergence of the dynamic structural adjustment process of RBF neural networks is still not well solved. Yu et al. [24] proposed an idea based on error correction, if the required performance is not achieved, the input corresponding to the point with the largest error after training is selected as the center of the next newly added neuron. This method can approximate the nonlinear function well using a more streamlined structure, but the method needs further research on the application to the actual process for information processing. Feng et al. used the particle swarm optimization (PSO) algorithm to optimize the center, width, and weights of the RBF network to achieve better modeling accuracy, but due to the global search capability of the algorithm, the training speed is slow and the algorithm is computationally large and complex, which is not conducive to real-time modeling [25,26].

In this paper, an information entropy-based dynamic tuning RBF (D-RBF) method is proposed to address the structural design and optimization of RBF neural networks when the effective input samples are small, and the information entropy-based RBF neural network is demonstrated to have good approximation ability for the prediction of nonlinear systems. The information entropy-based RBF neural network structure proposed in this paper is able to increase the activity and decrease the implicit layer neurons, which makes the D-RBF neural network more compact, has better approximation ability to nonlinear functions, high stability during training, and fast convergence. The theoretical validation of the convergence is given using the steepest descent learning algorithm [27], which proves the effectiveness of the method.

## 2. Information Entropy

The result of the quantitative measurement of uncertain information is called “information entropy”, which is an integration based on probability theory and numerical statistical analysis to express the degree of dispersion of a system, based on the entropy value to determine the evolutionary direction of the system. The multi-dimensional information is quantified and synthesized [28,29,30,31,32,33], which has broad implications for problems with complexity and uncertainty. The definition formula of information entropy is:

(1) $$H(x)=-\sum_{x\in \chi }p(x)\log p(x)$$

where *p(X)* is the probability of an event, and *H(X)* is a measure of the degree of discrepancy of uncertain information. In a RBF neural network, it can be used to measure the discreteness of a neuron activation data sample in the hidden layer.

The three properties of information entropy are: (1) monotonicity, the higher the probability of occurrence of events is, the lower the information entropy; (2) nonnegativity, information entropy is used to express the quantitative synthesis of information quantity of discrete system, where nonnegativity is a necessity; (3) accumulation, the synthesis of uncertainty quantity of system can be expressed as the sum of uncertainty measurement of discrete events.

(1) Mutual information, which shows the degree of intersection of event *X* and *Y*:

(2) $$I(X,Y)=\sum_{y\in Y}\sum_{x\in X}p(x,y)\log_{2}(\frac{p(x,y)}{p(x)p(y)})$$

where: *p(x)*, *p(y)* is the probability of an event, *p(x, y)* is the joint probability of event *X* and event *Y*, and *I(X, Y)* is the degree of crossover between event *X* and event *Y*.

The sum of the information quantity of event *X* and *Y* alone, where the sum of the information quantity of event *X* and *Y* occurs at the same time. When event *X* and *Y* are completely independent, the interactive information interaction information represents the intersection degree of event *X* and *Y.*

Joint entropy, which measures the uncertainty of the simultaneous occurrence of event *X* and event *Y*.

(3) $$H\left(X,Y\right)=-\underset{y\in Y}{\sum }\underset{x\in X}{\sum }p\left(x,y\right)\log\;p\left(x,y\right)$$

where *p(x, y)* is the joint probability of occurrence of event *X* and event *Y*, and *H (X, Y)* the measure of uncertainty of simultaneous occurrence of event *X* and event *Y*.

Conditional entropy, which measures the uncertainty of the occurrence of event *Y* at known event *X*.

(4) $$H\left(Y/X\right)=\underset{x\in X}{\sum }p\left(x\right)H\left(Y/x\right)$$

where *H(Y/X)* is a measure of uncertainty about the occurrence of event *Y* for a known event *X*, and *p(x)* is the probability of an event occurring.

**Figure 1:**
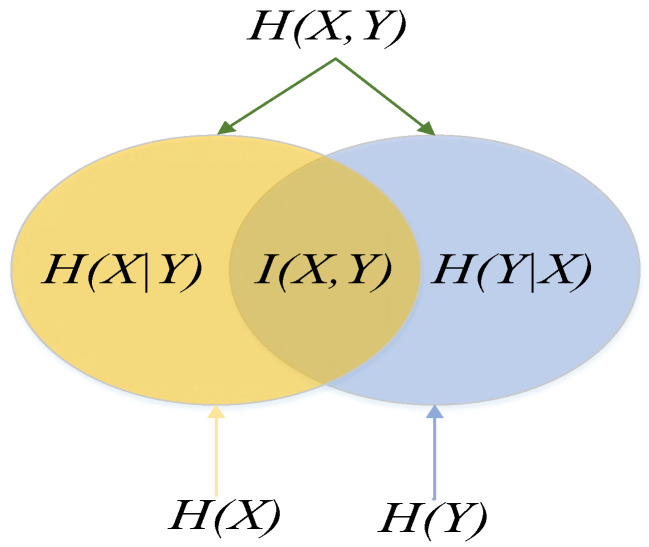
Event *X* and *Y* are connected by information entropy.

The relationship between the three is shown in Figure 1:

(5) I(X,Y)=H(Y)−H(Y/X)=H(X)−H(X/Y)

where *I(X, Y)* is the interaction information of event *X* and event *Y*.

The entropy of neuron *i* reflects the discrete degree of input samples, activated by neuron *i*. the larger the entropy is, the greater the discrete degree of input samples. The strength of the connection between the hidden layer neuron *i* and the output layer neuron is measured by information entropy. The interaction information will be smaller with the smaller impact of the discrete samples activated by neuron *i* on the output neuron *j*, and the disconnection can be considered. The sample of neuron *i* activation become more concentrated and the effect on the output neuron *j* become greater as the interaction information is greater. It is partitioning neuron *i*, splitting or disconnecting neurons according to the information entropy of the hidden layer neurons and output layer neurons to improve the efficiency of network operation [34].

## 3. RBF Neural Network

**Figure 2:**
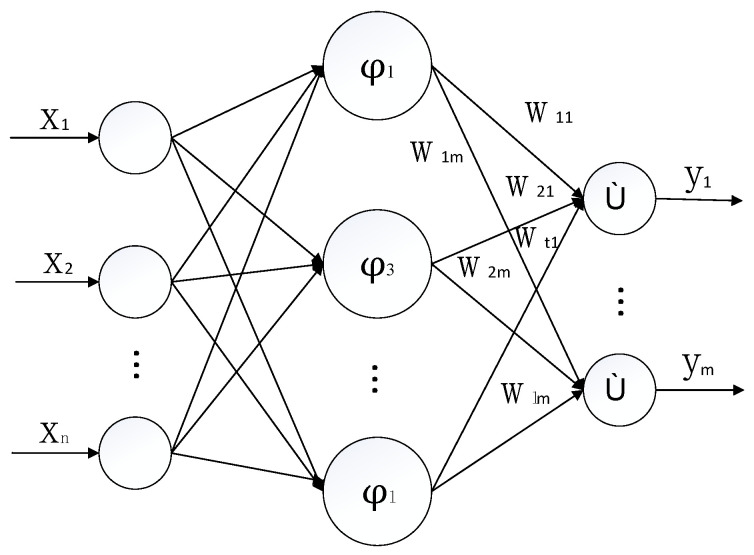
Structure of RBF neural network.

The process of RBF neural network is just like searching a curved surface which can match the sample data in high-dimensional space, and it uses the curved surface to interpolate the test data to find the unknown points [35]. The structure is shown in Figure 2, where *n* is the number of input data samples, and m is the number of hidden layer nodes. The output data of this experiment is a set of four-dimensional vectors:

(6) $$Y=\left[y_{1},y_{2},y_{3},y_{4}\right]$$

Select a group of test sample input data as:

(7) $$X=\left[x_{1},x_{2},\ldots x_{n}\right]$$

The center of the *i*-th hidden layer node is *C_i_*, and the activation function is:

(8) $$exp(-\frac{1}{2\delta_{i}^{2}}\|c_{i}-x_{i}\|b_{i}),i=1,2,\ldots n$$

where *b_i_* is the connection threshold between the *i*-th hidden layer neuron and the *j*-th output neuron, *c_i_* and *δ_i_* are the center and variance of node *i* of the hidden layer neuron, respectively, *x_i_* is the test sample input data.

The output of RBF neural network is as follows:

(9) $$Y_{j}=\overset{M}{\underset{i=1}{\sum }}\omega_{i}{}_{j}exp(-\frac{1}{2\sigma^{2}}\|c_{i}-x_{i}\|{}^{2}b_{i})$$

where ω*_ij_ (i = 1, 2…M, j = 1, 2, 3, 4)* is the connection weight of the *i*-th hidden layer and the *j* output unit, and *M* is the number of hidden layer neurons.

## 4. D-RBF Neural Network

**Figure 3:**
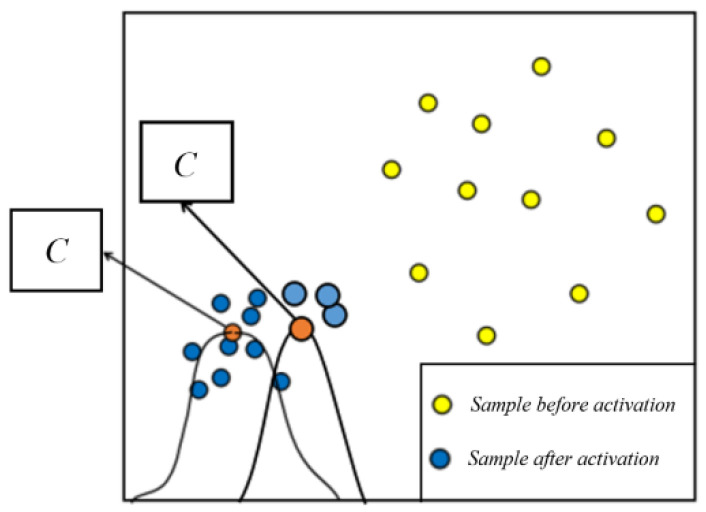
Dispersion of input samples after activation.

D-RBF chooses whether to divide neurons or not dynamically according to the connection strength of neuron nodes. Enough hidden layer neurons can ensure that the RBF network approaches any nonlinear function. In order to dynamically adjust the number of neurons in the hidden layer during training, the initial neuron node of this experiment is smaller than the sample dimension. By introducing entropy and information theory, the entropy of neuron node *i* can reflect the discreteness of the sample parameters, activated by the node. As shown in Figure 3, After activating the input sample, we find that the sample becomes more concentrated when the dispersion of node is smaller, and the entropy of node *i* also becomes smaller; on the contrary, the entropy of node *i* is larger. *C* is the central point of the activation function, the yellow point is the input sample before activation, and the blue point is the probability distribution of the input sample after activation.

**Figure 4:**
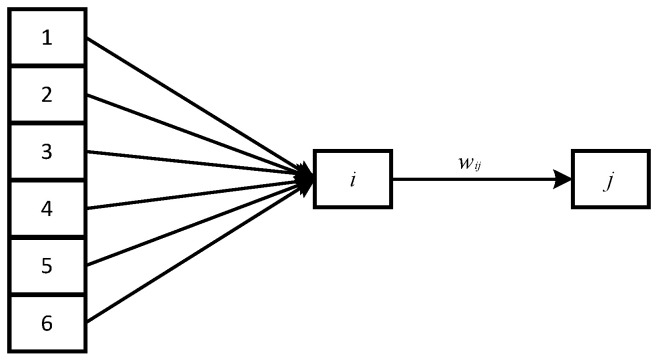
Sample data activation.

As shown in Figure 4, when samples *1, 2... p* are input to hidden layer *i,* the joint probability density function between node *i* and input samples is *f (p1, p2...p6, ai),* and the output of hidden layer neuron *i* is:

(10) $$a_{i}=\overset{6}{\underset{q=1}{\sum }}w_{ij}\phi_{qi}$$

where *W_ij_* is the connection weight of the *i*-th hidden layer to the *j*-th output unit, *φ* is the activation function of any hidden node.

**Figure 5:**
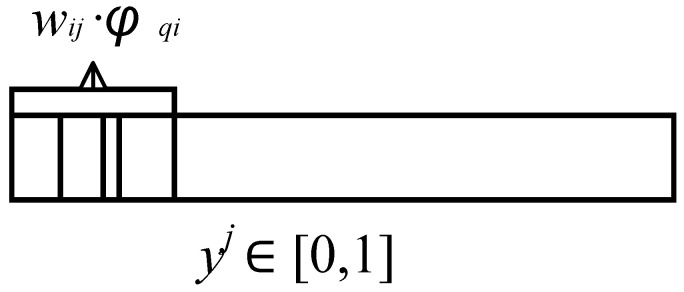
Composition of output neuron *j*.

As shown in Figure 5, the output *a_i_* of neuron *i* accounts for a small part of the total output *y_j_,* the joint probability density function of *M* hidden layer neurons and network output *y_j_*, is *f (a1, a2...a_M_, y_j_),* and the output of output neuron *j* is:

(11) $$y^{j}=\overset{M}{\underset{i=1}{\sum }}w_{ij}\phi \|c_{i}-p\|$$

where *W_ij_* is the connection weight of the *i*-th hidden layer to the *j*-th output unit, *c_i_* is the center of neuron node *i* in the hidden layer, p is the input sample, *M* is the number of neurons in the hidden layer, *φ* is the activation function of any hidden node, *y^j^* is the entropy of the network output.

The entropy of node *i* represents the degree of information dispersion among the *p* samples. The degree of comprehensive activation of neuron *i* to input samples increases when the entropy is smaller, leading to the greater attraction of neuron node *i* to *p* samples [36]. The information interaction between node *i* and node *j* is in the state of high load calculation, and small data error will lead to incalculable prediction results. In order to ensure the stability of network training, node *i*, the entropy of node *j* represents the difference of contribution of *M* neurons in the hidden layer to the total output. The large entropy indicates that the contribution of *M* neurons in the hidden layer to the output is large. The hidden layer neuron *i* with small contribution should be disconnected from the output layer neuron *j.*

The entropy of neuron node *i* can be expressed as:

(12) $$H\left(i\right)=-\frac{1}{p}\log_{2}\frac{\phi_{1i}}{\sum_{q=1}^{p}\phi_{qi}}-\frac{1}{p}\log_{2}\frac{\phi_{2i}}{\sum_{q=1}^{p}\phi_{qi}}-\ldots -\frac{1}{p}\log_{2}\frac{\phi_{6i}}{\sum_{q=1}^{p}\phi_{qi}}$$

where *φ* is the activation function of any hidden node and *p* is the total number of samples.

The entropy of network output *y^j^* is expressed as:

(13) $$H\left(y^{j}\right)=-\frac{1}{M}\log_{2}\frac{\sum_{q=1}^{p}w_{1j}\cdot \phi_{q1}}{y^{j}}-\frac{1}{M}\log_{2}\frac{\sum_{q=1}^{p}w_{2j}\cdot \phi_{q2}}{y^{j}}-\ldots -\frac{1}{M}\log_{2}\frac{\sum_{q=1}^{p}w_{6j}\cdot \phi_{q6}}{y^{j}}$$

where *W_ij_* is the connection weight of the *i*-th hidden layer to the *j*-th output unit, *M* is the number of neurons in the hidden layer, *φ* is the activation function of any hidden node, and *p* is the total number of samples, *y^j^* is the entropy of the network output.

The contribution of output of neuron *i* to total output of network is given as:

(14) $$H\left(i,y^{j}\right)=-\frac{1}{M}\log_{2}\frac{\sum_{q=1}^{p}w_{ij}\cdot \phi_{qi}}{y^{j}}$$

where *W_ij_* is the connection weight of the *i*-th hidden layer to the *j*-th output unit, *M* is the number of neurons in the hidden layer, *φ* is the activation function of any hidden node, and *p* is the total number of samples, *y^j^* is the entropy of the network output.

The adjustment of the connection relationship between node *i* and node *j* depends on the connection strength of node *i* and node *j I(i, j)*

(15) $$I\left(i,j\right)=H\left(i\right)-H\left(i/j\right)=H\left(i\right)-\frac{H\left(i,y^{j}\right)}{H\left(y^{j}\right)}$$

**Figure 6:**
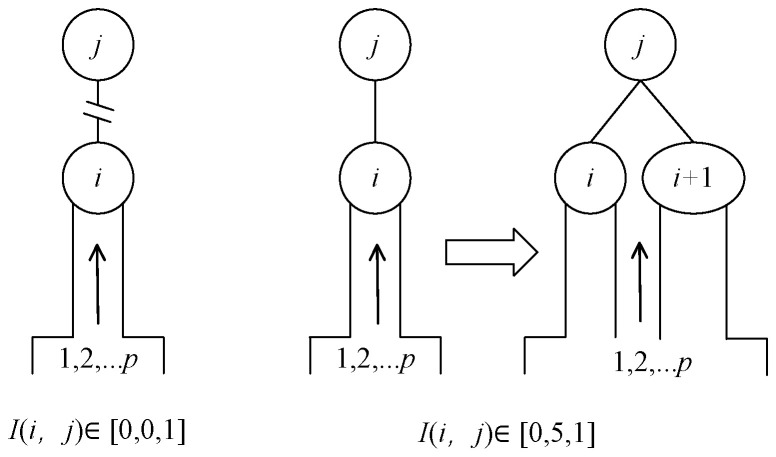
Division or disconnection of neuron I.

Neuron *i* and *j* are independent of each other. When *I (i, j)* is large, the neuron node *i* and output node *j* have strong information interaction ability. Hidden layer node *i* has great attraction to *p* input samples, and node *i* may be in calculation state of high load [36]. At this time, node *i* is divided into 2 neurons, as shown in Figure 6.

## 5. Proof of Convergence

### Dynamic Adjustment of the Network Structure 

**Figure 7:**
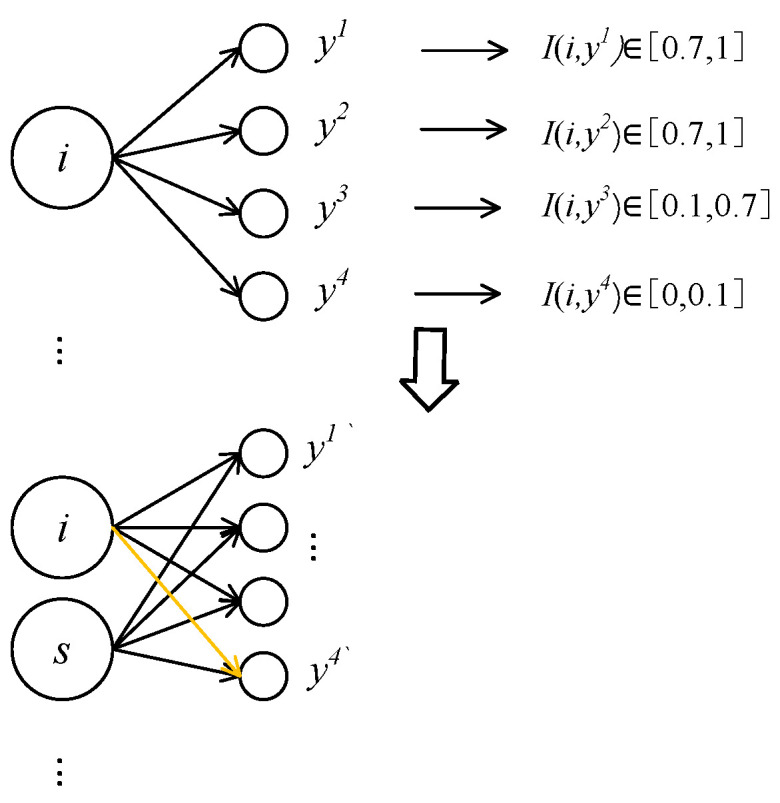
Neuron *i* splits into two, disconnects *i* from *y*^4^.

When the network is training the *M*-group samples, the number of hidden layers is *n*, and the error is *e(k) = d(k) − y(k), d(k)* is the expected output, *y(k)* is network output. The information association strength *I(i, j)* between the hidden layer node and the output layer node is calculated according to Equation (11). As shown in Figure 7, node *i* is divided into two nodes. The center point of the new node *j* has the same variance and the connection weight becomes half of the original value.

Before and after the structural adjustment, the original and new output are:

(16) $$y^{1}+y^{2}+\ldots +y^{4}=\overset{4}{\underset{j=1}{\sum }}w_{ij}\phi \|c_{i}-p\|$$

(17) $$\begin{array}{l} y^{1^{\backslash }}+y^{2}{}^{\backslash }+\ldots +y^{4}{}^{\backslash }=\overset{4}{\underset{j=1}{\sum }}\frac{w_{ij}}{2}\phi \|c_{i}-p\|-w_{i4}\phi \|c_{i}-p\|+\overset{4}{\underset{j=1}{\sum }}\frac{w_{sj}}{2}\phi \|c_{i}-p\|\\ =\overset{4}{\underset{j=1}{\sum }}w_{ij}\phi \|c_{i}-p\|-w_{i4}\phi \|c_{i}-p\| \end{array}$$

where *W_ij_* is the connection weight of the *i*-th hidden layer to the *j*-th output unit, *φ* is the activation function of any hidden node before and after structural adjustment, *c_i_* is the center of neuron node *i* in the hidden layer, *p* is the total number of samples, and *y^j^* is the entropy of the network output.

Because the connection strength, between neuron *i* and *y*^4^ output, is less than 0.1, the neuron *i* has a low activation degree to *p* samples, a large sample dispersion degree, and very small impact on output, which can be ignored. When network structure is adjusted, the connection between hidden layer neuron and *y^4^* is ignored. So *y(k) = y′(k)*, the error *e(k) = d(k) − y(k) = d(k) − y′(k).*

During the training process, the structure of RBF neural network is adjusted, and the output error *e(k)* of the network is not changed, which increases the convergence speed of the average error *E*.

(18) $$E=\frac{1}{2p}\overset{p}{\underset{k=1}{\sum }}e^{2}\left(k\right)$$

where: *e* is the network output error and *p* is the number of samples

In the process of training, by splitting the neurons in the hidden layer with high connection strength, the connections with low connection strength are disconnected. Under the premise of constant output value, the stability of the output value of the network is guaranteed and the collection is improved convergence speed [27].

## 6. Prediction of Regression Value of UAV Flight Status

### 6.1. Establishment and Analysis of UAV Model

**Figure 8:**
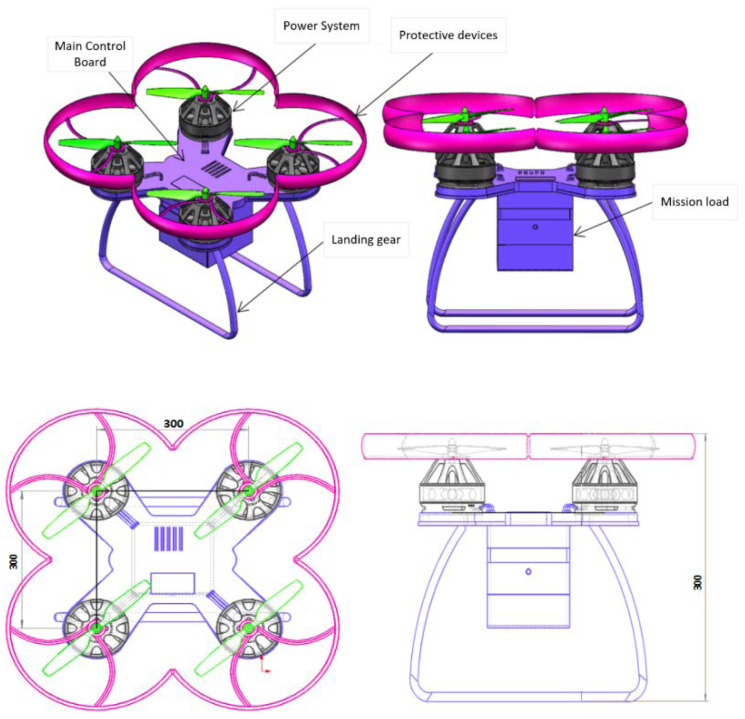
Schematic diagram of rotary wing logistics UAV.

The logistics UAV consists of a power system, main control board, protective devices, mission load and landing gear. The motion state of the drone refers to vertical motion, pitch motion, transverse motion, and roll motion. To put it simply, vertical motion refers to ascending and descending, pitch motion refers to forward and backward motion, transverse motion refers to left and right motion, and roll motion refers to changing direction. The four-rotor motors control the attitude angle in flight, and the change of motor speed controls the change of attitude angle. Four propellers are distributed in a “x” cross pattern. The lift generated by the rotor supports the UAV’s own weight and completes various flight maneuvers. The rotor generates lift as well as air resistance, so the choice of wing shape is critical to the amount of lift and drag. The model of the quadrotor transport UAV is shown in Figure 8.

**Figure 9:**
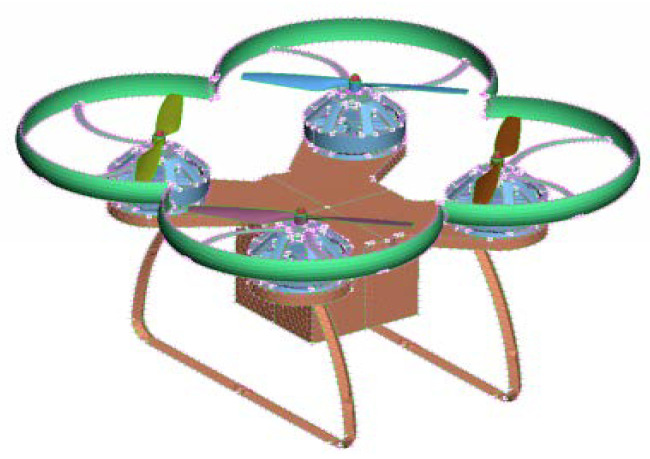
Finite element model of four rotor aircraft.

In this experiment, the finite element model of the logistics UAV in ANSYS software is shown in Figure 9. In order to control the number of grids and ensure the quality of grids, the small structures, such as bolts and wires, are cleaned up, and the calculation domain of UAV outflow field is constructed by topology. Because the rotation region of propeller is the key area of calculation, the grid is encrypted to ensure the quality of calculation. Finally, the triangular mesh is 43,752, and the number of volume mesh is 1,156,169. After checking, the mesh quality meets the calculation requirements.

### 6.2. Flow Field Analysis during Flight

**Figure 10:**
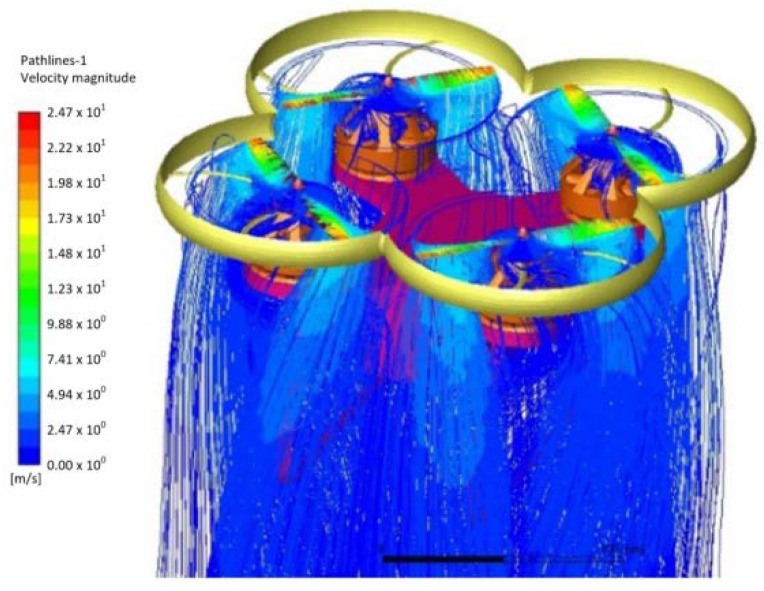
Flight flow field.

**Figure 11:**
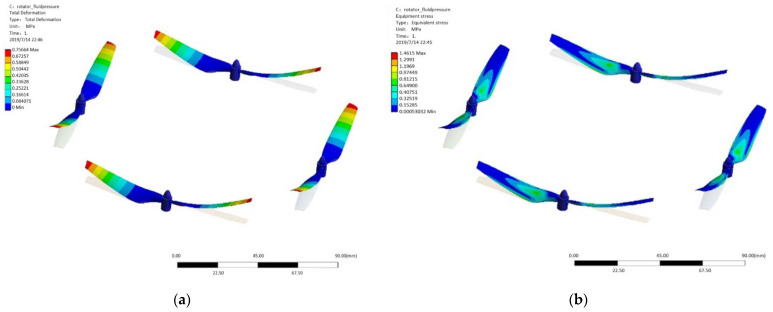
Stress cloud map (**a**) and deformation cloud map (**b**) of rotor under lift and self-weight.

It can be seen from the streamline diagram (Figure 10) that, in the flight process, the downwash flow generated by the rotor is spiraling downward perpendicular to the rotating plane without considering the wind interference, the flow velocity is gradually reduced, and the lift is generated. At the same time, the airflow interference between the rotors is relatively small, which has little impact on the working efficiency of a single rotor. The flow field distribution is reasonable. According to the calculation results, the torque of a single rotor is 0.008N/m. The maximum lifting force is 0.61 kg, which meets the requirements of working conditions (fuselage 0.6 kg, motor 0.1 × 4 kg, load 0.6–1 kg). Within this range, the rotor + motor selection is reasonable. The maximum deformation (0.76 mm) of the UAV rotor is located at the tip of the rotor blade. The maximum stress is 1.4 MPa, which is less than the allowable stress 25 MPa of the plastic used in this paper. From the safety margin formula:

*MS* = σ_s_/(σ_max_ * f) − 1

where *MS* is the safety margin; *σ_s_* is the maximum permissible stress; *σ_max_* is the calculated maximum stress; *f* is the safety factor, which is 1.5 in this paper, and the calculated safety margin at the maximum stress of the rotor blades is 16.85, which is greater than zero. The material safety of rotor is reasonable. In addition, according to cloud Figure 11, the deformation and stress of the structure, at the installation hole in the center, are small, with high safety margin and no damage.

### 6.3. Flight Test

**Figure 12:**
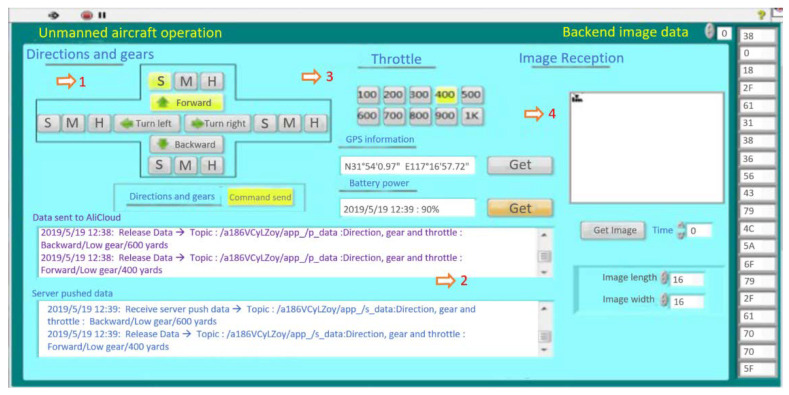
User interface connects with cloud platform through mqtt protocol to control Internet of things UAV.

Three types of UAV (the maximum tension produced by battery with motor and propeller is known) are tested. In order to obtain the flight status of the logistics UAV under different weight ratios and different output tensions, in the experiment, the logistics UAV is equipped with different cargo loads to fly at different flight speeds, recording the maximum flight time, and making judgments and records on the flight stability within the maximum flight time. As shown in Figure 12, when the maximum pull force is known, different throttle can obtain different output pull force. The user interface sends commands to control the takeoff, landing and flight of the Internet of Things (IOT) logistics UAV. Part 1 is direction control and flight speed control; Part 2 is user interface sending instructions to cloud platform. After receiving the message, the cloud platform sends the message to the UAV. Part 3 is the quantitative output throttle to control the output pull of logistics UAV.

Table 1 records the flight status of logistics UAVs with different mass ratios and output tensions over the range time. According to the test results of four rotor propeller and the aerodynamic calculation analysis of UAV, the lift drag ratio of logistics UAV was ***L/D*** = 8.5. According to the barometric altimeter of logistics UAV, we calculate the time required to rise to 5 m, $t=\sqrt{2s/a}$, and the output tension, ***F = ma***. When the logistics UAV flies at a constant speed at a height of 5 m, the flight distance within the flight time is recorded, and the flight speed is obtained ***v = s/t.*** According to many groups of data, the flight state was recorded. The data was used to train RBF neural network, and the key nodes were selected for flight experiment. 

According to the experimental input/output data, the flight state of logistics UAV is a highly nonlinear change process. RBF neural network is suitable for regression fitting of the flight state of logistics UAV to obtain the best load ratio of UAV.

### 6.4. Normalization of Experimental Data

Input variables of the experiment are maximum pulling force, output pulling force, flight resistance, load capacity, flight speed and maximum flight time, and the input vector is:

(19) $$x=[F_{\max},f_{out},f_{for},m_{\max},v_{ave},t_{\max}]$$

The contents and expressions of the six parameters are inconsistent. The elements in the sample set are scaled to the known maximum and minimum values, and the original data is normalized. The conversion formula is shown in Equation (20). As shown in Table 2, after normalization, the process of finding the optimal output layer weight will become smooth, and the convergence speed will be faster and more stable.

(20) $$x_{i}=x-x_{i}^{min}/x_{i}^{max}-x_{i}^{min}$$

### 6.5. Simulation Experiment

**Figure 13:**
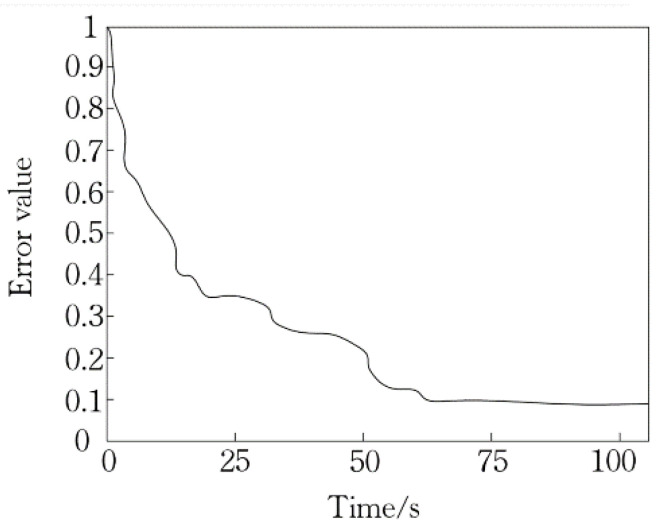
Error variation in convergence process.

**Figure 14:**
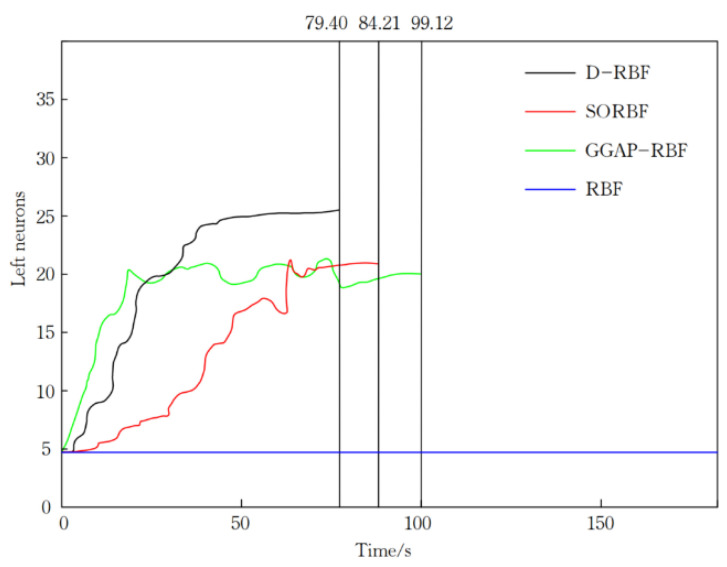
The number of neurons in hidden layer during network training.

In this paper, the maximum pulling force, output pulling force, flight resistance, load, flight speed and maximum flight time of logistics UAV are used as input variables of the D-RBF neural network training. The output of network training is a set of four-dimensional vectors to represent the UAV’s flight state in the maximum time. 21 sets of data are selected as training samples and 10 sets of data are selected as test samples. In the training process, the convergence time of D-RBF and the number of hidden layer nerves are shown in Figure 13 and Figure 14. In Figure 13 as the number of training per second increases, the error becomes smaller and smaller, and when it exceeds 75 time/s. The change of error is no longer obvious. 

In Figure 14, as the number of training per second increases, the left neurons of D-RBF first increase sharply, then tend to grow slowly, and its convergence time is 79.40; the left neurons of SORBF first increase sharply, then fall slightly in the middle, then tend to grow slowly, and its convergence The convergence time of SORBF is 84.21; the left neurons of GGAP-RBF rise sharply first, then fall slightly in the middle, and then tend to grow slowly, and the convergence time is 99.12; the left neurons of RBF do not change, and the convergence time is 236.72.

The evaluation index of network output error is established as:

(21) $$e=\sum_{k=1}^{4}\left|d_{p}(k)-e_{p}(k)\right|$$

where *d_p_(k)* is the expected output and *e_p_(k)* is the network output error.

**Figure 15:**
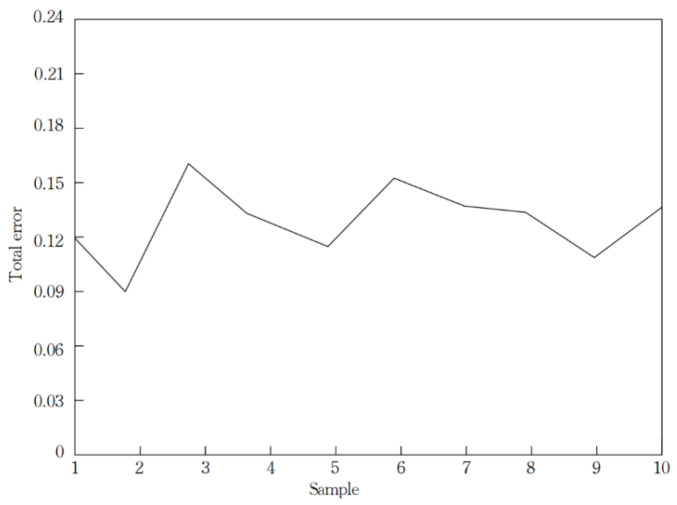
Error values of ten groups of test samples.

The error between the expected value and the output value of ten groups of test samples is shown in Figure 15, In Figure 15 the total error changes significantly as the number of samples increases, but the total error lies in the interval of 0.09 to 0.18. The average performance of D-RBF, SORBF, RBF and GGAP-RBF, trained ten times respectively, is shown in Table 3.

The simulation results show that D-RBF can well predict the regression value of the nonlinear system of the logistics UAV. In Table 3, the number of neurons in the final neural network of D-RBF is 24, which is higher than that of RBF, SORBF and GGAP-RBF, the detection error of D-RBF is 0.0138, which is much smaller than that of RBF, SORBF and GGAP-RBF, and the convergence time of D-RBF is 79.40 times, is the smallest. This indicates that the average training time of D-RBF is better than the other three neural network algorithms, and the detection error is the smallest.

In this paper, we take the flight status prediction of UAV as a case study, and propose an information entropy-based RBF neural network structure adjustment and optimization method for the problem of inaccurate convergence that occurs when the effective training samples are small. It solves the problem of dynamic adjustment of RBF neural network structure in training, and D-RBF convergence analysis is provided. Compared with other RBF neural networks, the following conclusions are drawn. The conclusion is as follows:

(1) The D-RBF neural network does not depend on the initial structure of the network. It can dynamically adjust the number of neurons and disconnect the weakest connection, according to the connection strength of hidden layer neurons and output layer neurons. It can respond in real time.
(2) The entropy of hidden layer neuron and output layer neuron is calculated. The output information of hidden layer neuron and the connection strength between hidden layer neuron and output layer neuron are measured, and the mathematical expression is given to realize the dynamic adjustment of network structure.
(3) By experimentally comparing the performance differences among D-RBF, SORBF, RBF, and GGAP-RBF, the convergence speed of the average error is accelerated and D-RBF is proved to have good convergence performance.
(4) The present D-RBF neural network solves the regression prediction of the flight state of the logistics UAV, providing guidance for the research of the flight stability performance of the logistics UAV under different loads and flight speeds.

## Figures and Tables

**Table 1:** Experimental data of logistics UAV.

Number	Pulling Force (N)	Output Pull (N)	Flight Resistance (N)	Fuselage Weight (kg)	Flight Speed (m/s)	Flight Time (min)	Flight Condition Weights (Excellent, Moderate, Poor, Noisy)
1	3.2	1.76	0.21	1.1	3.58	9.21	(0.90, 0.05, 0.04, 0.01)
2	3.2	1.92	0.23	1.2	3.9	8.46	(0.87, 0.1, 0.03, 0)
3	3.2	2.08	0.24	1.4	4.14	6.24	(0.68, 0.1, 0.0.08, 0.1)
4	3.2	2.24	0.26	1.0	4.72	7.18	(0.88, 0.1, 0.02, 0)
5	3.2	2.4	0.28	1.7	4.10	3.76	(0.58, 0.08, 0.1, 0.24)
6	3.2	2.88	0.34	1.4	4.65	5.28	(0.74, 0.08, 0.06, 0.12)
7	3.2	3.04	0.36	1.1	6.08	5.46	(0.84, 0.13, 0.03, 0)
8	3.2	3.2	0.38	2.0	5.32	2.19	(0.21, 0.13, 0.17, 0.49)
9	4.0	2.6	0.31	1.0	4.37	7.92	(0.91, 0.06, 0.03, 0)
10	4.0	2.8	0.33	1.2	5.01	6.31	(0.89, 0.07, 0.04, 0)
11	4.0	3.2	0.38	1.6	5.98	5.41	(0.84, 0.09, 0.04, 0.03)
12	4.0	3.4	0.40	1.8	6.08	3.85	(0.71, 0.05, 0.03, 0.21)
13	4.0	3.6	0.42	2.1	6.01	2.77	(0.41, 0.15, 0.06, 0.38)
14	4.0	3.8	0.45	1.6	6.88	4.08	(0.69, 0.21, 0.06, 0.04)
15	4.0	4.0	0.47	1.4	7.04	4.14	(0.76, 0.16, 0.06, 0.02)
16	4.8	3.12	0.37	1.7	5.12	7.36	(0.91, 0.04, 0.05, 0)
17	4.8	3.36	0.40	1.9	6.08	6.01	(0.79, 0.09, 0.04, 0.08)
18	4.8	3.6	0.42	2.8	4.75	3.56	(0.40, 0.01, 0.08, 0.51)
19	4.8	4.08	0.48	2.1	5.63	4.28	(0.51, 0.25, 0.1, 0.14)
20	4.8	4.56	0.54	1.4	7.52	4.83	(0.87, 0.08, 0.05, 0)
21	4.8	4.8	0.56	1.9	7.49	4.27	(0.71, 0.18, 0.07, 0.04)

**Table 2:** Six nodes and experimental results after the normalization process.

Normalized Processing Nodes	Maximum Pulling Force	Output Pull	Flight Resistance	Weight Capacity	Flight Speed	Endurance Time
1	0.32	0.176	0.021	0.11	0.358	0.921
2	0.32	0.192	0.023	0.12	0.39	0.846
3	0.32	0.208	0.024	0.14	0.414	0.624
4	0.32	0.224	0.026	0.10	0.472	0.718
5	0.32	0.240	0.028	0.17	0.410	0.376

**Table 3:** Average performance comparison.

Function	Algorithm	Expected Error	Detection Error	Final Network	Convergence Time
Regression fitting	D-RBF	0.01	0.0138	24	79.40
	RBF	0.01	0.0326	6	236.72
	SORBF	0.01	0.0163	20	84.21
	GGAP-RBF	0.01	0.0142	19	99.12
